# Molecular Genetic Characteristics of the *Hoxc13* Gene and Association Analysis of Wool Traits

**DOI:** 10.3390/ijms25031594

**Published:** 2024-01-27

**Authors:** Hongxian Sun, Zhaohua He, Fangfang Zhao, Jiang Hu, Jiqing Wang, Xiu Liu, Zhidong Zhao, Mingna Li, Yuzhu Luo, Shaobin Li

**Affiliations:** Gansu Key Laboratory of Herbivorous Animal Biotechnology, Faculty of Animal Science and Technology, International Wool Research Institute, Gansu Agricultural University, Lanzhou 730070, China; sunhx@st.gsau.edu.cn (H.S.); hezh@st.gsau.edu.cn (Z.H.); zhaofangfang@gsau.edu.cn (F.Z.); huj@gsau.edu.cn (J.H.); wangjq@gsau.edu.cn (J.W.); liuxiu@gsau.edu.cn (X.L.); zhaozd@gsau.edu.cn (Z.Z.); limn@gsau.edu.cn (M.L.); luoyz@gsau.edu.cn (Y.L.)

**Keywords:** hair follicle, *Hoxc13* gene, spatiotemporal expression, SNPs, sheep

## Abstract

Homobox C13 (Hoxc13) is an important transcription factor in hair follicle cycle development, and its deletion had been found in a variety of animals leading to abnormal hair growth and disruption of the hair follicle system. In this study, we used immunofluorescence, immunohistochemistry, real-time fluorescence quantitative PCR (RT-qPCR), and Kompetitive Allele-Specific PCR (KASP) genotyping to investigate molecular genetic characteristics of the Hoxc13 gene in Gansu alpine fine-wool sheep. The results revealed that Hoxc13 was significantly expressed during both the anagen and catagen phases (*p* < 0.05). It was found to be highly expressed predominantly in the dermal papillae and the inner and outer root sheaths, showing a distinct spatiotemporal expression pattern. Two single nucleotide polymorphisms (SNPs) in the exon 1 of Hoxc13, both the individual locus genotypes and the combined haplotypes were found to be correlated with wool length (*p* < 0.05). It was determined the mutations led to changes in mRNA expression, in which higher expression of this gene was related with longer wool length. In summary, this unique spatiotemporal expression pattern of the Hoxc13 gene may regulate the wool length of Gansu alpine fine-wool sheep, which can be used as a molecular genetic marker for wool traits and thus improve the breed.

## 1. Introduction

The hair follicle is a complex microscopic organ that undergoes cyclic transformations of anagen, catagen, and telogen phases [1]. A very important condition in the cycle of hair follicle development is the regenerative system of the hair follicle. This regenerative action of the hair follicle depends mainly on a unique interaction between the epithelium and the mesenchyme [2,3]. In recent years, several molecular signals such as fibroblast growth factor, transforming growth factor-β, WNT signaling pathway, sonic hedgehog, neurotrophins, and homeobox, and their interactions in the hair follicle cycle have been identified [4,5,6]. The hair cycle is tightly controlled by autonomous and external molecular signals, and defects in the hair cycle may lead to skin diseases such as alopecia [7].

Homeobox (HOX) proteins contain an evolutionarily conserved DNA-binding homeobox domain. Several members of HOX have been detected in different subregions of the hair follicle during the early stages of mouse hair development [8,9], Hoxc13 is a key member of the HOX family and plays an important role in regulating cell proliferation, differentiation and apoptosis [8,10,11]. In mice, over expression of *Hoxc13* led to hair brittleness, and this phenomenon was also observed in mouse *Hoxc13* mutants [8,12]. In humans, mutations in *Hoxc13* are associated with complete hair loss in pure hair and nail ectodermal dysplasia (PHNED) [13]. The abnormal phenotypes of hair and nails in transgenic mice [12] overexpressing *Hoxcl3*, as well as in *Hoxc13* mutant mice [8], *Hoxc13* mutant pigs [14], and humans [15], suggest that the *Hoxc13* gene may regulate the expression of the genes that are associated with the formation of hair and nail such as hair- or nail-specific keratin and keratin-associated protein (KAP) genes.

Given the potential regulatory role of the *Hoxc13* gene in hair follicle development and hair growth, characterization of the gene and it association with wool traits is of significance for the development of molecular markers for improving wool traits. In this study, we investigated *Hoxc13* in Gansu alpine fine-wool sheep using immunofluorescence, immunohistochemistry, sanger sequencing, Kompetitive Allele-Specific PCR (KASP), and RT-qPCR, and we analyzed the association between *Hoxc13* genotypes, haplotypes, and wool traits. Therefore, this study evaluated using a candidate gene approach to provide some support for subsequent studies.

## 2. Results

### 2.1. AOD Values of Hoxc13 in Different Stages of Skin Follicle Cycle Development

**Figure 1:**
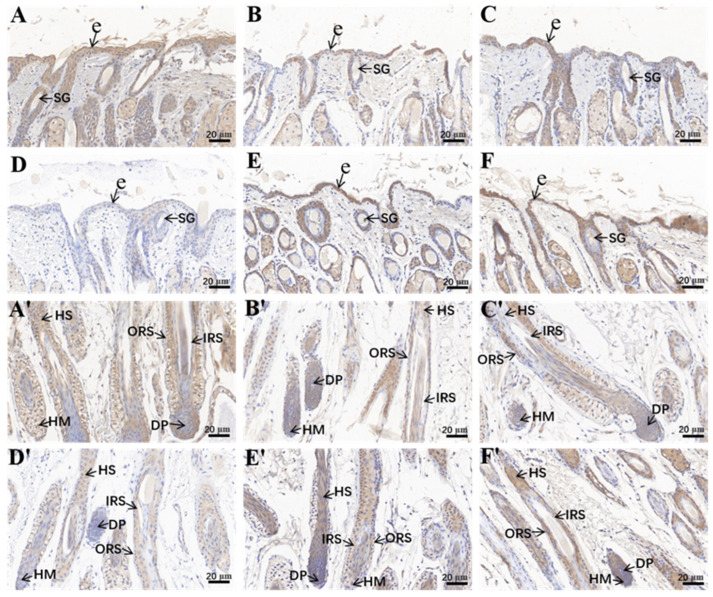
Immunohistochemical staining of Hoxc13 at different stages of skin hair follicle cycle development (40×). **A**(**A**′)–**F**(**F′**): six groups of immunohistochemical staining at day 1, 30, 60, 90, 180, and 270, in that order. (**A**,**A′**) are the same sample from the same period, (**A**) is the upper part of the hair follicle, (**A′**) is the lower part of the hair follicle. All similarly numbered samples that follow are the same sample. ORS: outer root sheath; IRS: inner root sheath; DP: dermal papilla; HM: hair matrix; HS: hair shaft; e: epidermis; SG: sebaceous glands.

**Figure 2:**
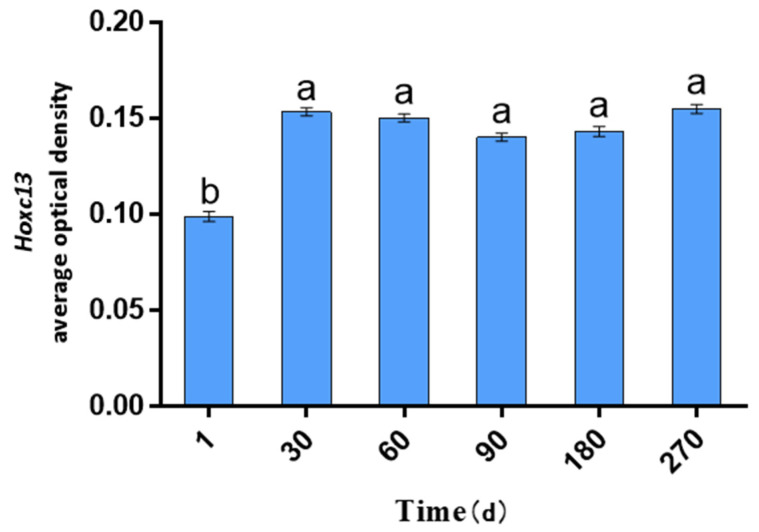
AOD values of Hoxc13 at different stages of hair follicle cycle development in the skin. The data are presented as mean ± standard error (S.E.). Different letters indicate significant differences between ages (*p* < 0.05).

Hoxc13 protein expression was counted in immunohistochemical sections of skin at different stages of hair follicle cycle development, and multiple sites were selected for counting the optical density of stable expression and calculating it using the AOD statistic (AOD = IOD/Area) (Figure 1). The results of AOD showed that Hoxc13 protein was expressed in skin at different stages, and the protein level changed significantly at different stages of hair follicle cycle development. The AOD of Hoxc13 in 1-day-old skin was the lowest among the six periods (*p* < 0.05; Figure 2).

### 2.2. Distribution and Localization of Hoxc13 in Different Stages of Hair Follicle Cycle Development in the Skin

**Figure 3:**
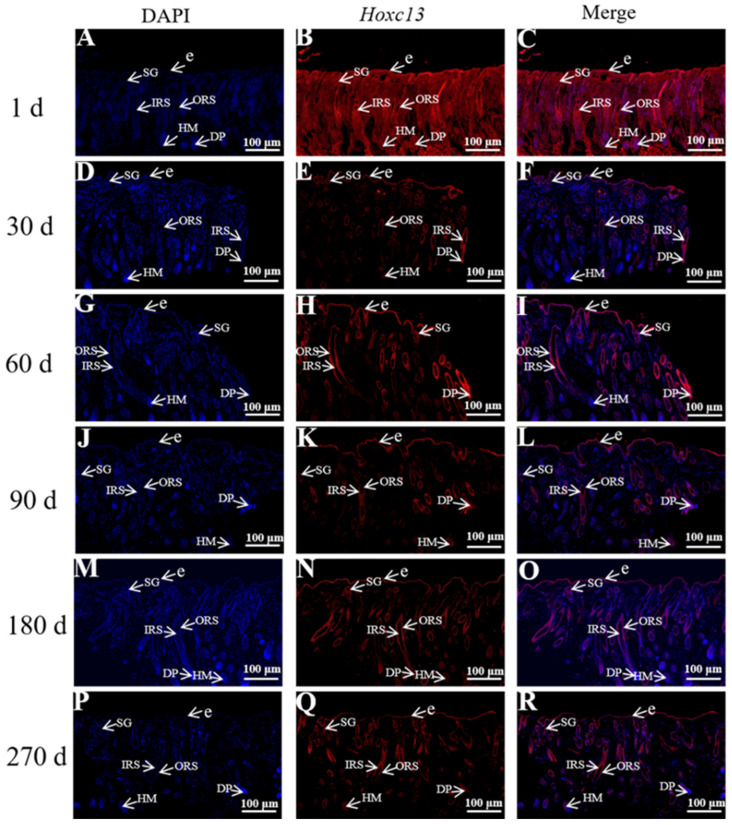
Immunofluorescence staining of Hoxc13 at different stages of skin follicle cycle development (10×). (**A**–**C**): 1 day; (**D**–**F**): 30 days; (**G**–**I**): 60 days; (**J**–**L**): 90 days: (**M**–**O**): 180 days; (**P**–**R**): 270 days. The blue tissue in the figure shows DAPI-labeled nuclear fluorescence staining and the red tissue shows fluorescence staining of Hoxc13. ORS: outer root sheath; IRS: inner root sheath; DP: dermal papilla; HM: hair matrix; e: epidermis; SG: sebaceous gland.

The immunofluorescence technique was used to analyze the protein expression of Hoxc13 in the skin at different periods of time, and the intensity of expression was scored positively (Figure 3). Positive scoring results showed Hoxc13 expressed in all six stages of skin hair follicle cycle development, and showed high expression in the dermal papillae and the inner and outer root sheaths, weak expression in hair medulla, and medium or strong expression in the corneum (Table 1). According to the results of different stages of hair follicle development, Hoxc13 may play an important role in the hair follicle cycle, which is regulated mainly through the dermal papillae and the inner and outer root sheaths.

### 2.3. PCR Amplification Product Electrophoresis, Product Sequencing Results, and SNP Loci Detection Results

**Figure 4:**
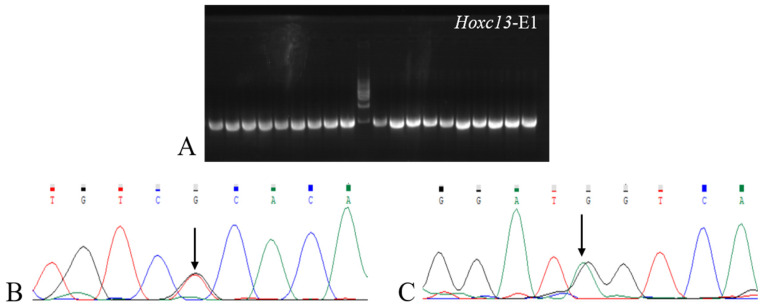
PCR amplification and sequencing results of *Hoxc13*-E1 in Gansu alpine fine-wool sheep. (**A**) is the PCR band plot of *Hoxc13*-E1 amplification product. (**B**,**C**) are *Hoxc13* sequencing results. The overlapping peaks indicated by arrows are SNPs mutation sites.

**Figure 5:**
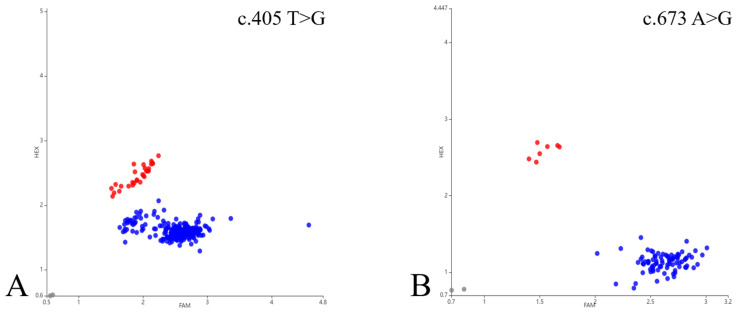
Results of KASP genotyping analysis of two positions of *Hoxc13* gene in Gansu alpine fine-wool sheep. Horizontal and vertical coordinates for the two joints recognized by mutant base signaling, respectively. FAM is blue, HEX is green, and FAMHEX is red. Red and blue dots in (**A**) indicate *TG* and *TT* genotypes, respectively, and red and blue dots in (**B**) indicate *AG* and *AA* genotypes, respectively. Gray dots in plots (**A**,**B**) represent unrecognized signals.

Sequencing of the first exon of *Hoxc13* in 20 Gansu alpine fine-wool sheep revealed a total of two variant sites (Figure 4). A Blast comparison of *Hoxc13* sequencing results identified two SNPs at c.405 and c.673, which were analyzed by KASP in 315 samples, whereas five samples failed to be identified, and in the 310 samples that were successfully identified, these SNPs were found to show two genotypes, *TG* and *TT*, and *AG* and *AA*, respectively (Figure 5).

### 2.4. Genotype Frequencies, Gene Frequencies, and Population Genetic Diversity at Each Locus of the Hoxc13 Gene

KASP genotyping was performed on all DNA samples, and the genotype frequencies and gene frequencies of the two SNPs loci were analyzed in the Gansu alpine fine-wool sheep population (Table 2). Two SNPs were detected in the Hoxc13 gene at c.405 (G/T) and c.673 (A/G) with the latter altering the amino acid from serine to glycine. The highest genotype frequency was observed for TT (0.8613) at c.405 (G/T) locus and AA (0.8613) at c.673 (A/G) locus.

The population genetic diversity of the two SNPs loci was counted in the Gansu alpine fine-wool sheep population (Table 3), and both SNP loci were in Hardy–Weinberg equilibrium in the sheep population (*p* > 0.05), and the frequency of homozygotes is higher than that of heterozygotes. These two SNP provided low polymorphic content (PIC < 0.25).The association analysis of different genotypes of two SNPs of the Hoxc13 gene with the traits of Gansu alpine fine-wool sheep wool was performed (Table 4), and the results showed that, two SNPs of the Hoxc13 gene were strongly associated with wool length (*p* < 0.05). The sheep with genotype TG and AG have a longer MSL than TT and AA, respectively (*p* < 0.05). The data for all wool traits were tested with the assumption of normal distribution and all conformed to a normal distribution.

### 2.5. Haplotype Analysis in the Hoxc13 Gene of Gansu Alpine Fine-Wool Sheep

Linkage disequilibrium analysis of the *Hoxc13* gene showed that a haplotype block could be constructed for the 2 SNPs loci of the *Hoxc13* gene, and the 2 SNPs were in weak linkage (D′ = 0.72, R^2^ = 0.518). As can be seen from Table 5, after the haplotype construction of the *Hoxc13* gene, four haplotypes with frequency > 0.01 were constructed, i.e., H1 (*TA*, 0.913), H2 (*GG*, 0.051), H3 (*GA*, 0.018), and H4 (*TG*, 0.018), and the combination of four haplotypes of the *Hoxc13* gene showed a total of two haplotype combinations (H1H1, H1H2) with frequency > 0.03. As shown in Table 6, the association analysis of these two haplotype combinations with wool traits indicated that the two different haplotype combinations of the *Hoxc13* gene were significantly (*p* < 0.05) associated with wool length, where the wool length of H1H2 individuals was significantly higher than that of HIH1 (*p* < 0.05).

### 2.6. Results of RT-qPCR for Different Genotypes of Two SNPs in the Hoxc13 Gene of Gansu Alpine Fine-Wool Sheep

**Figure 6:**
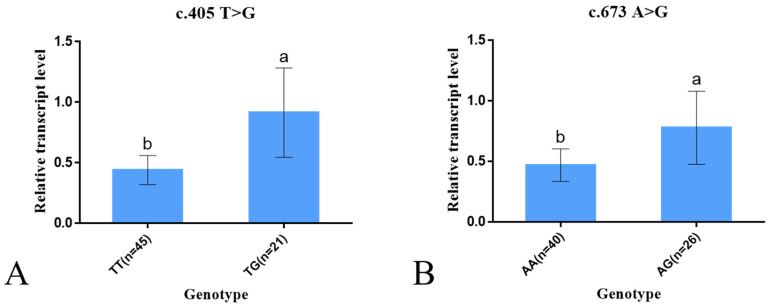
Results of RT-qPCR for different genotypes of two SNPs in the *Hoxc13* of Gansu alpine fine-wool sheep. The results are expressed as mean ± standard error (S.E.), and different letters are indicated as significant (*p* < 0.05). (**A**) is the result of mRNA expression for both genotypes of mutation c.405 and (**B**) is the result of mRNA expression for both genotypes of mutation c.673.

RT-qPCR study of different genotypes of two SNPs of the *Hoxc13* gene in Gansu alpine fine-wool sheep revealed (Figure 6). The c.405 locus *TG* genotype showed significantly higher expression lever than *TT* genotype (*p* < 0.05); c.673 locus *AG* genotype was significantly high expressed than *AA* genotype (*p* < 0.05). Two SNPs of the *Hoxc13* gene were expressed at higher levels in heterozygotes than in homozygotes.

## 3. Discussion

### 3.1. Protein Expression and Localization Study of Hoxc13 in Skin Tissues of Different Ages

Hair follicle growth and development is a complex long-term physiological process regulated by a variety of physical factors and signaling pathways, and gene expression is one of the determining factors affecting wool growth [16,17]. Therefore, in this study, we investigated the expression of Hoxc13 protein in skin tissues of Gansu alpine fine-wool sheep at different periods, and found that Hoxc13 was expressed at different periods, and showed strong temporal and spatial expression characteristics, with the lowest expression at 1 day of age (telogen), which also indicated that Hoxc13 may play an important role in the development of hair follicles, especially during the anagen and catagen phase. Studies have shown that Hoxc13 knockout in rats, rabbits, and pigs found that the animals appeared to have less hair and no hair [16]. Another study found that Hoxc13 was expressed in the epidermis, outer root sheath, inner root sheath, and hair shaft of secondary hair follicles, but showed a high expression in the outer root sheath [18]. Hoxc13 was found to be expressed in the epidermis and outer root sheaths and correlated with the skin thickness of the Hexi cashmere goat [19], whereas in the Tibetan cashmere goat, Hoxc13 was found to be expressed mainly in the outer root sheath and hair shaft [20]. In addition, the expression of Hoxc13 was found to have an effect on hair follicle activity, which has a stimulatory effect on hair follicle development [18]. In this study, we found that Hoxc13 showed high density strong positive expression in the outer root sheath and inner root sheath in the different stages of skin follicle cycle development, which is consistent with previous studies. Combined with previous studies, it is suggested that Hoxc13 regulates hair follicle growth and development and periodicity through the outer and inner root sheaths. In addition, this study also found that Hoxc13 also showed a high expression in the dermal papillae, which has not been reported in other studies. At the base of the hair follicle, there are cells called papilla cells, which play an important role in hair growth and renewal because they can secrete various factors and thus regulate the surrounding tissues [21,22]. It was found that the dermal papillae have an important role in the hair follicle development cycle because of their function of receiving and sending signals. Therefore, this study found that Hoxc13 regulated hair follicle cycle development and hair growth through the outer root sheath, inner root sheath, and dermal papillae in Gansu alpine fine-wool sheep.

### 3.2. Identification of Hoxc13 Polymorphisms and Association Analysis of Wool Traits

*Hoxc13* plays an important role in the morphogenesis and cyclic development of hair follicles, and has a great influence on the formation of hair traits. And it was found that *Hoxc13* gene deletion [13] and mutation [23] cause dysplasia of pure hair and nail ectodermal. Therefore, in a study of *Hoxc13* polymorphisms, the results showed that the Blast comparison of *Hoxc13* sequencing of exon 1 results revealed two SNPs. One of the SNPs from *A* to *G* at the c.673 site resulted in a missense mutation of the amino acid serine to glycine. It has been found that missense mutations tend to occur in the coding region, so that biological traits are altered as a direct result of the occurrence of missense mutations, and that alterations in the base sequences cause changes in the initially translated protein sequences, which ultimately allow for changes in the function of the proteins, leading to the development of many diseases [24]. Although the other mutation site, c.405, is synonymous and does not affect the amino acid sequence of the protein it translates, it has been found that the synonymous mutation affects the efficiency or accuracy of mRNA shearing, the miRNA binding site, or the translation efficiency, as well as the structure and quantity of the expressed protein product, which we should not be ignored [25,26]. An association analysis of the two *Hoxc13* SNPs genotypes with wool traits revealed that both were significantly associated with wool length (*p* < 0.05). Also, to further investigate the relationship between the two SNPs, the two points were found to be weakly interlocked and the haplotype combinations were also found to be significantly correlated with the mean sample length, which in turn further supports the results of the genotype–wool trait association analysis.

To further investigate the effect of mutation on *Hoxc13* mRNA expression, mRNA expression of different genotypes was measured and found to be higher in heterozygotes than in the observed homozygotes. The higher expression of *Hoxc13* for the *TG* and *AG* genotypes is associated with longer wool fibers, compared to the *TT* and *AA* genotypes, suggesting a positive correlation between the *Hoxc13* expression and wool fiber growth. Although we did not detect the *GG* genotype at the two SNP sites, it is safe to speculate that individuals with *GG* genotypes have higher expression levels. It was shown that in a study on the role of bone morphogenetic protein (BMP) in nail differentiation, a high expression of *BMP5* in nail fibroblasts was observed by *BMP5* treatment of cultured human nail matrix keratin-forming cells (NMK), and also, increased expression of *Hoxc13* was observed [27]. Studies on mice have found that the injection of recombinant *Hoxc13* polypeptide (rhHoxc13) at the telogen phase significantly promoted hair growth and induced initial hair growth progression [28]. Ectodermal dysplasia-9 (ED-9) is a congenital disorder with clinical manifestations of hypotrichosis and nail dystrophy, and *Hoxc13* is the pathogenic gene for ED-9 [29]. In a study in pigs, it was found that when *Hoxc13* was knocked down, hair follicles showed various abnormal phenotypes, such as reduced hair follicles and abnormal hair all over the body [14]. The clinical features of knockout mice all exhibit generalized hairlessness and nail abnormalities, while in the mouse study, the gene ends of *Hoxc13* were truncated, which is similar to the clinical features of knockout mice with purified *Hoxc13* [30].

It has been found that the level of mRNA expression is affected to some extent by intronic mutations [31]. It has also been shown that missense and intronic mutations cause changes in gene mRNA expression and that such changes are significantly associated with these two mutations [32]. The occurrence of synonymous mutations can have an effect on production traits, which has also been reported in several studies [33,34,35,36]. Studies have demonstrated that keratin, a target gene of *Hoxc13*, is critically regulated by *Hoxc13* and also has a regulatory role in hair follicle development, and mutations in *Hoxc13* have been shown to cause hair defects in mice and human patients [8,37,38]. Meanwhile, seven SNPs in *Hoxc13* have been identified so far in human studies to cause PHNED in patients with clinical manifestations of complete hair loss and nail dysplasia [13,39]. And two of these SNPs belong to missense mutations (c.812A>G and c.929A>C) leading to PHNED [38,40]. In a study on velvet goats, two SNPs (c.812A>G and c.929A>C) were found in the homologous structural domain of *Hoxc13*, which could lead to the loss of regulatory function of *Hoxc13* on keratin, but did not affect the protein expression of *Hoxc13* [41]. Therefore, this also suggests that changes in alleles caused by synonymous and missense mutations could potentially make changes in the sequence of DNA and RNA, further affecting gene transcription and mRNA expression. Interestingly, a 27.6 kb homozygous microdeletion was found in the first exon of *Hoxc13* in a female patient from Afghanistan, and this deletion resulted in a significant reduction in *Hoxc13* mRNA levels in skin tissue [13]. Similarly, in a study of knockout pigs, it was found that *Hoxc13* mutations resulted in reduced or even absent *Hoxc13* mRNA expression, and that the expression levels of the *Hoxc13* downstream genes *Foxn1*, *Krt85*, and *Krt35* were similarly reduced [14]. And this is consistent with the findings of this study that *Hoxc13* mutations will result in changing of mRNA expression of *Hoxc13*. The same reduction in expression may present with hairlessness symptoms. Thus, this also demonstrates that mutations do cause changes in mRNA expression and ultimately affect production traits; i.e., mutations in the *Hoxc13* gene would cause changes in *Hoxc13* mRNA expression, thus further affecting wool length. Increasing the length of wool will increase its textile value. However, the present study found that genotypes with less distribution within the population (*GT* and *AT*) were more important for wool length, leading to longer wool. This may due to the sheep herd being small with very few rams. Next, it is necessary to expand the population to further validate the experimental results.

In summary, the present study discusses the *Hoxc13* gene, and finds a unique and strong expression pattern both temporally and spatially. We found that *Hoxc13* gene is highly expressed in the anagen and catagen of hair follicle development, and we further localized the expression sites and found that the *Hoxc13* gene is highly expressed in the dermal papillae and the inner and outer root sheaths of the hair follicle. Thus, we can infer that *Hoxc13* has an important role in the anagen to realize the maintenance of hair follicle development and wool. Then, a further study found that the first exon of *Hoxc13* has two SNPs, and these two mutation sites were significantly correlated with the length of the wool, and its mutation leads to allelic changes that will result in changes in the expression of mRNA. This also suggests that this unique spatiotemporal expression pattern of *Hoxc13* may regulate the trait of wool length.

## 4. Materials and Methods

### 4.1. Animal Samples

All of the sheep used in this research came from the same herd in Tianzhu Tibetan Autonomous County, Gansu Province, China, to ensure they had the same environmental conditions.

Three newborn healthy Gansu alpine fine-wool sheep ewes with similar weight randomly selected. At 1, 30, 60, 90, 180, and 270 days after birth, a skin tissue of 5 cm^2^ area was taken from the posterior edge of the scapula of each lamb, and the sample was cleaned with distilled water. A portion of the sample was frozen rapidly in liquid nitrogen and stored at −80 °C. The other part of the sample was fixed in 4% paraformaldehyde and stored at 4 °C. We used 2% lidocaine for local anesthesia on the posterior edge of the scapula of Gansu alpine fine-wool sheep, and then treated with penicillin (Hebei Cheng sheng tang Animal Pharmaceutical Co., Ltd., Shijiazhuang, China) and other drugs to prevent wound infection after sampling was completed, and bandaged with gauze.

A total of 315 Gansu alpine fine-wool sheep of the same age (1 year old) and sex (female) were selected. Blood was collected from all sheep using sodium citrate anticoagulation tubes, then stored at −20 °C and brought back to the laboratory for subsequent experiments. At the same time, wool collection was performed within 5 cm^2^ of the scapula for subsequent analysis. Wool traits were measured by the New Zealand Pastoral Measurements LTD. (Ahuriri, Napier, New Zealand).

Sixty-six one-year-old Gansu alpine fine-wool sheep ewes were selected at June (hair follicles are in the growth phase), and their skin tissues were taken from the posterior edge of the scapula within an area of 5 cm^2^ of skin. The samples were washed with distilled water, stored in a liquid nitrogen tank, and brought back to the laboratory at −80 °C. These skin samples were used for analyzing mRNA expression of *Hoxc13*.

### 4.2. Immunohistochemical Analysis

Refer to previous research for detailed steps [42], in brief, paraffin sections were routinely dewaxed to water and washed with phosphate-buffered saline (PBS) before antigen repair with citric acid antigen repair buffer (pH = 6.0). The sections were incubated with 3% hydrogen peroxide solution at room temperature for 25 min in darkness to eliminate the activity of endogenous peroxidase. The slices were dripping with the proportional *Hoxc13* rabbit primary antibody (bs-13599r) prepared with PBS, and were treated overnight in the box at 4 °C. The sections were spun dry and the corresponding secondary antibody was added, and then freshly prepared Diaminobenzidine (DAB) was added to the sections for color development, which was positive (brown and yellow), and the color development was terminated by using running water. Steps were as follows: Tap water fully rinsed, counterstained, dehydrated; transparent, sealed film; microscope examination, image acquisition, and analysis.

### 4.3. Immunofluorescence Analysis

Refer to previous research for detailed steps [42]; in brief, paraffin sections were routinely dewaxed to water, and antigen repair was carried out with ethylene diamine tetra-acetic acid (EDTA) antigen repair buffer (pH = 8.0), and then bovine serum albumin (BSA) was added dropwise to block for 30 min. The slices were dripping with the proportional *Hoxc13* rabbit primary antibody (bs-13599r) prepared with PBS, and were treated overnight in the box at 4 °C, and then the corresponding secondary antibody was added by drop and incubated for 50 min at room temperature. After the sections were slightly dried, DAPI dihydrochloride (DAPI) dye solution was added to the rings, and the slices were incubated for 10 min at room temperature in the dark. Autofluorescence quencher was added into the ring for 5 min, and then washed with water for 10 min. The sections were slightly dried and sealed with anti-fluorescence quench sealer. The sections were observed and images were collected under a fluorescence microscope.

### 4.4. PCR Amplification and Genotyping

*Hoxc13* is the causative gene for PHNED, and two missense mutations have been found in humans that lead to hairlessness [38,40]. The first exon was selected for the study because it was found to have a missense mutation in the first exon of the *Hoxc13* gene predicted by the Ensemble website for sheep. Primers of 200 bp before and after this exon were designed using Oligo7 software (Beijing Huanzhong Reach Technology Co., Ltd., Beijing, China) (Table 7). Twenty Gansu alpine fine-wool sheep were selected for PCR amplification, and the amplification products were later sequenced. Primer synthesis and sequencing were performed at Sangon Biotech Co., Ltd. (Shanghai, China) to further determine the location of SNPs. KASP typing was performed on all samples using an enzyme marker with fluorescence resonance energy transfer (FRET) capability, and the analysis was performed by Gentides Biotechnology (Wuhan, China).

### 4.5. RT-qPCR Analysis

Trizol method (Thermo Fisher Scientific Co., Ltd., Shanghai, China) was used for the extraction of total RNA from the collected skin samples. The purity and concentration of the extracted RNA were then determined using ultraviolet spectrophotometer and subsequently stored at −80 °C. RNA reverse transcription was performed using the Prime Script^TM^ RT kit (Nanjing Novizan Biotechnology Co., Ltd., Nanjing, China) according to the manufacturer’s instructions, and the cDNA was then stored at −20 °C. *β-actin* was selected as the internal reference gene. All primers and PCR information are given in Table 1. RT-qPCR was carried out using Biosystems QuantStudio^®^6 Flex (Thermo Lifetech, Waltham, MA, USA) and SYBR Green Pro Taq HS qPCR Kit (Accurate Biology, Changsha, China). To ensure the authenticity and reliability of the results, we used three biological replicates and four technical replicates for each period.

### 4.6. Measurements and Statistical Analysis

Immunohistochemistry and immunofluorescence were scanned and imaged using PANNORAMIC (3DHISTECH Ltd., Budapest, Hungary) panoramic slice scanner. The cumulative optical density (IOD) values of six positive fields in each slice were measured by Image-Pro 6.0 software (Media Cybernetics Inc., Rockville, MD, USA). The corresponding positive pixel Area (Area), and AOD = IOD/Area was calculated. Positive scoring was performed on five selected sites within three fields of view per section (Note: +, Weak expression; ++, Medium expression; +++, Strong expression; ++++, High expression). For fluorescence quantification, the raw data were first processed using the Excel 19.0 software package and then calculated after the commonly used −2^ΔΔCT^ method [43]. The genotype frequencies, allele frequencies, homozygosity (Ho), heterozygosity (He), effective allele numbers (Ne), and polymorphism information content (PIC) of *Hoxc13* gene SNPs were counted, and the *X*^2^ test for Hardy–Weinberg equilibrium detection. Correlation of *Hoxc13* SNPs genotypes with wool traits was analyzed using *t*-tests. All results of this study were analyzed using the SPSS 22.0 (SPSS Inc., Chicago, IL, USA) statistical software package, and the results are expressed as mean ± standard error (S.E.), and different letters are indicated as significant (*p* < 0.05). SPSS 22.0 Kolmogorov–Smirnova (K-S test) was used to test the assumption of normal distribution of wool trait data.

## 5. Conclusions

In conclusion, this study showed that (1) the *Hoxc13* gene was mainly expressed in the outer root sheath, inner root sheath, and dermal papillae of Gansu alpine fine-wool sheep to achieve the regulation of hair follicle cycle; and (2) it was found that two SNPs in exon 1 of the *Hoxc13* gene were significantly associated with wool length, and the mutations led to changes in mRNA expression, and there was a positive correlation between the Hoxc13 expression and wool fiber growth. It can be used as a candidate gene or molecular genetic marker to improve Gansu alpine fine-wool sheep and increase economic benefits.

## Figures and Tables

**Table 1:** Positive intensity of Hoxc13 in various parts of skin tissue at different ages.

Days	Corneum	Inner Root Sheath	Outer Root Sheath	Hair Medulla	Dermal Papillae
1	+++	++++	++++	+	++++
30	++	++++	++++	+	++++
60	++	++++	++++	+	++++
90	++	++++	++++	+	++++
180	++	++++	++++	+	++++
270	++	++++	++++	+	++++

Note: +, Weak expression; ++, Medium expression; +++, Strong expression; ++++, High expression.

**Table 2:** Genotype frequency and gene frequency of 2 SNPs of *Hoxc13* gene.

Gene	SNP Site	Number	Genotype Frequency (Number)		Allele Frequency	
*Hoxc13*	c.405 T > G	310	*TT*	*TG*	T	G
			0.8613 (267)	0.1387 (43)	0.9306	0.0694
	c.673 A > G	310	*AA*	*AG*	A	G
			0.8613 (267)	0.1387 (43)	0.9306	0.0694

**Table 3:** The population genetic diversity of 2 SNPs of *Hoxc13* gene.

Gene	SNP Site	Ho	He	Ne	PIC	Hardy–Weinberg *p*-Value
*Hoxc13*	c.405 T > G	0.8708	0.1292	1.1484	0.1209	*p* > 0.05
	c.673 A > G	0.8708	0.1292	1.1484	0.1209	*p* > 0.05

**Table 4:** Effect of different genotypes of *Hoxc13* gene SNPs on wool traits.

Gene	SNP Site	Trait	Genotype (Number)	
*Hoxc13*	c.405 T > G		*TT* (267)	*TG* (43)
		Mean Fiber Diameter (μm)	22.182 ± 0.172	22.384 ± 0.408
		Mean Staple Length (mm)	80.019 ± 1.297 ^b^	94.114 ± 3.606 ^a^
		Mean Fiber Curve (n/mm)	105.359 ± 0.802	105.514 ± 2.490
		Comfort Factor (%)	88.241 ± 0.780	85.890 ± 3.120
		Fiber Shift	12.245 ± 0.257	13.533 ± 0.881
		Strength (cN/dT)	14.332 ± 0.416	14.847 ± 1.329
	c.673 A > G		*AA* (267)	*AG* (43)
		Mean Fiber Diameter (μm)	22.274 ± 0.174	21.887 ± 0.372
		Mean Staple Length (mm)	79.717 ± 1.256 ^b^	95.941 ± 3.986 ^a^
		Mean Fiber Curve (n/mm)	105.359 ± 0.791	109.169 ± 3.478
		Comfort Factor (%)	87.793 ± 0.790	90.375 ± 3.163
		Fiber Shift	12.433 ± 0.252	11.518 ± 1.156
		Strength (cN/dT)	14.353 ± 0.410	14.381 ± 1.610

Note: Different letters indicate significant differences.

**Table 5:** Frequency of haplotype and haplotype combination of SNPs locus reconfiguration in *Hoxc13* gene.

Haplotype	SNP1	SNP2	Frequency	Haplotype Combination	Frequency
H1 (TA)	T	A	0.913	H1H1	0.836
H2 (GG)	G	G	0.051	H1H2	0.047
H3 (GA)	G	A	0.018		
H4 (TG)	T	G	0.018		

**Table 6:** Effect of *Hoxc13* gene reconfiguration haplotype on wool traits.

Haplotype Combination	Mean Fiber Diameter (μm)	Mean Staple Length (mm)	Mean Fiber Curve (n/mm)	Comfort Factor (%)	Fiber Shift	Strength (cN/dT)
H1H1	22.161 ± 0.168	79.970 ± 1.272 ^b^	105.143 ± 0.799	88.403 ± 0.767	12.296 ± 0.263	14.293 ± 0.427
H1H2	22.332 ± 0.617	100.794 ± 4.062 ^a^	112.740 ± 4.611	85.600 ± 4.611	12.808 ± 1.376	16.551 ± 2.157

Note: Different letters indicate significant differences.

**Table 7:** Primer sequences and annealing temperatures used for PCR and RT-qPCR.

Gene	GenBank Accession No.	Primer Sequence (5′–3′)	Product Length (bp)	Annealing Temperature (°C)	Application
*Hoxc13*	ENSOARG00020018195	F: GGCGGTGGAAACACCAGGAG	1007	60	PCR
		R: TCCATCTGCAGCCCAGCAAAG			
*Hoxc13*	AY_266017.1	F: GATAGTCAGGTGTACTGCTC	132	60	RT-qPCR
		R: CTGCGTACTCCTTCTCTAGC			
*β–actin*	NM_001009784	F: AGCCTTCCTTCCTGGGCATGGA	113	60	
		R: GGACAGCACCGTGTTGGCGTAGA			

## Data Availability

The authors affirm that all data necessary for confirming the conclusions of the article are present within the article, figures, and tables.
